# Trends and factors contributing to health facility delivery among adolescent women in Ethiopia: multivariate decomposition analysis

**DOI:** 10.1186/s12905-022-02069-2

**Published:** 2022-12-02

**Authors:** Asaye Alamneh Gebeyehu, Dejen Gedamu Damtie, Chalachew Yenew

**Affiliations:** Department of Public Health, College of Health Science, Debre Tabor University, Debre Tabor, Ethiopia

**Keywords:** Health facility delivery, Trend, Adolescent women, EDHS, Ethiopia

## Abstract

**Background:**

Although an increase in health facility delivery in Ethiopia over time, adolescent women giving birth at health facilities is still low. Health facility delivery is crucial to improving the health of women and their newborns' health by providing safe delivery services. We aimed to examine the trend change and identify factors contributing to health facility delivery in Ethiopia.

**Methods:**

We analyzed the data on adolescent women obtained from three Ethiopian Demographic and Health Surveys. A total of weighted samples were 575 in 2005, 492 in 2011, and 378 in 2016. Data management and further statistical analysis were done using STATA 14. Trends and multivariate decomposition analysis were used to examine the trends in health facility delivery over time and the factors contributing to the change in health facility delivery.

**Results:**

This study showed that the prevalence of health facility delivery among adolescent women in Ethiopia increased significantly from 4.6% (95% CI 3.2–6.7) in 2005 to 38.7% (95% CI 33.9–43.7) in 2016. Decomposition analysis revealed that around 78.4% of the total change in health facility delivery over time was due to the changes in the composition of adolescent women and approximately 21.6% was due to the changes in their behavior. In this study, maternal age, place of residency, wealth index, maternal education, frequency of ANC visits, number of living children, and region were significant factors contributing to an increase in health facility delivery over the study periods.

**Conclusion:**

The prevalence of health facility delivery for adolescent women in Ethiopia has increased significantly over time. Approximately 78.4% increase in health facility delivery was due to adolescent women’s compositional changes. Public health interventions targeting rural residents and uneducated women would help to increase the prevalence of health facility delivery.

## Background

Maternal and child death has prominent public health coy6ncerns in low and middle-income countries, particularly in Sub-Saharan Africa (SSA) countries [1, 2]. Pregnancy and childbirth-related complications are the leading causes of death for women and newborn babies in lower-income countries, particularly Ethiopia [3–5]. More than 1.2 million adolescent women died each year from all preventable diseases including pregnancy and childbirth complications [6]. Pregnancy and childbirth-related compactions such as unsafe abortion, infection, Hemorrhage, obstructed labor, and hypertension during pregnancy are a massive burden for adolescent women’s mortality [7, 8].

The World Health Organization (WHO) recommend that health facility delivery as one of the key strategies to prevent maternal and new child mortality [9]. Giving birth at medical health facilities (government or non-government health facilities) is crucial to prevent childbirth complications and improve health outcomes [10, 11]. Providing safe delivery services supported by skilled birth attendance is essential to ensure maternal and fetal health outcomes [12, 13]. Although health facility delivery is the key strategy to improve maternal and neonatal health outcomes, many adolescent women in developing countries give birth at home without the assistance of trained personnel [14].

Early marriage is one of the most traditional harmful practices, contributing to abnormal health conditions and the incidence of adolescent pregnancy [15, 16]. Ethiopian official legal proclaimed that the age of marriage for women is more than 18 years [15], but most people have practiced early marriage, and 58% of the girls get married before 18 years, and also 35% of them become pregnant at the age of 15–19 [17, 18]. Several studies done across the world have shown that adolescent and young women are less likely to access adequate and essential maternal health care services such as antenatal care booking, postnatal care visits, and giving birth in health facilities than adults or older women (women more than the age of 19 years) [19–21]. To reduce the maternal mortality ratio and improve pregnancy outcomes, create awareness about the benefits of delivering the child in a health facility for both women’s and newborn babies’ health and scale up the rate of childbirth in the health facilities [22, 23].

Giving birth in the health facility with the assistance of skilled birth attendants is essential to achieve the Sustainable Developmental Goals (SDGs) and improve the maternal and newborn babies' health [24, 25]. There is inadequate usage of health care services in rural communities than in urban communities due to access to health facilities, lack of appropriate facilities, and distance of health facilities in the community [26–28]. According to the EDHS 2016 report, around 70% and 40% of urban and rural women had given birth in the health facility. The overall prevalence of health facility delivery among childbearing women in Ethiopia has increased from 5% in 2000 EDHS to 26% in 2016 EDHS [13, 29].

Previous studies conducted around the world and in Ethiopia have identified several factors affecting the use of health facility delivery. Factors associated with health facility delivery were socio-demographic and economic variables, obstetric-related factors, and accessibility of health facilities [12, 30–38]. Evidence showed that no previous studies have been conducted on health facility delivery among adolescent women using decompositional analysis. Despite progress in health facility delivery over time, there was low health facility delivery in Ethiopia. Therefore, this study aimed to examine the trends and identify factors contributing to health facility delivery in Ethiopia using multivariate decompositional analysis. This finding will help concerning bodies to design strategies and interventions for adolescent women to improve maternal health care services utilization.

## Methods

### Study design and setting

This study used three consecutive Ethiopian Demographic and Health Surveys (EDHS 2005, EDHS 2011, and EDHS 2016). According to the 2007 Population and Housing Census (PHC) of Ethiopia, the complete list of enumeration areas (EAs) was created as a census frame. At the time of the survey, Ethiopia is sub-divided into nine regional states and two metropolitan cities. The regional states are Tigray, Afar, Amhara, Oromia, Somali, Benshangul-Gumuz, Southern Nations, Nationalities, and Peoples' Region (SNNPR), Gambela, and Harari. The metropolitan cities were Addis Ababa and Dire Dewa. A stratified cluster sampling was implemented to select EDHS samples of enumeration areas (EAs) in each stratum independently. Each region in Ethiopia was stratified separately into urban and rural areas, yielding 21 sampling strata. The sample size in each stage were selected through probability proportion to cluster size. The samples used in these EDHSs were selected in two stages. In the first stage, among the total 540 EAs (395 in rural areas) for EDHS 2005, 624 EAs (437 in rural areas) for EDHS 2011, and 645 EAs (443 in rural areas) for EDHS 2016 were chosen proportional to cluster size.

A household listing procedure was performed in all selected EAs, and the list of households serves as a sampling frame for selecting individuals in the second stage. In the second stage, 28 households per cluster were selected with an equal probability of systematic selection from established household lists. More information on sampling techniques and methods is available from the EDHS report [18, 39, 40].

The data sets were obtained from children's recode data or kids file (KR file) of EDHS 2005, 2011 and 2016; accessed from the DHS program website (http://www.measuredhs.com). A total of 575 in 2005, 492 in 2011, and 378 adolescent women in 2016 were involved in this study.

### Study population and period

The study population was all adolescents (women aged 15–19 years) who gave birth in the five years preceding the survey in selected Enumeration Areas (EAs) in Ethiopia in this study. The data collection period for three consecutive 2005, 2011, and 2016 EDHSs were conducted from February 2000 to May 2000, April 27 to August 30, 2005, December 27 in 2010 to June 3 in 2011, and from January 18 to June 27 in 2016, respectively[41–43].

### Study variables

#### Outcome variable

The outcome variable for this study was health facility delivery among adolescent women. The response variable for the i^th^ adolescent women who give birth in the health facility is a random variable. The binary response Yi was coded as one if adolescent women gave birth at health facilities (government, private, and non-government health facilities); zero if they did not give birth at a health facility.

#### Independent variables

The variables used for this study were considered independent variables. The socio-demographic variables and obstetric factors. The socio-demographic variables were maternal age, place of residency, marital status (single, married/living together, widowed/separated, others), religion (Orthodox, Muslim, Protestant, Other), working status, wealth index status (poor, middle, rich), maternal educational status and region. Indeed, the number of living children in a household (no child, first, two and above), frequency of ANC visits (no ANC visits, < 4 ANC visits, and at least 4 ANC visits), distance to health facility, and problem seeking permission to go healthcare services were considered obstetric factors. The region is categorized as large central region (Tigray, Amhara, Oromia, and SNNPs), metropolitan geographical regions (Harari, Addis Ababa, and Dire Dawa), and semi-peripheral region (Tigray, Amhara, Oromia, and SNNP) based on the geographical features, consistent with previous study in Ethiopia [44, 45].

### Data management and analysis

Data extraction, cleaning, coding, and statistical analysis were performed using STATA 14 software. We have extracted the relevant variables from the dataset in this study. Before doing any statistical analysis, the samples used in this study were weighted using probability sampling weight, which adjusted the non-response rate to restore the national representative sample and get valid statistical estimates. Appending the dataset on adolescent women obtained from 2005, 2011, and 2016 EDHSs together after extracting relevant variables for trends and multivariate decomposition analysis.

### Trend and decomposition analysis

The trend period was divided into three phases; first phase (2005–2011), third phase (2011–2016), and fourth phase (2005–2016) to show the change in the prevalence of health facility delivery over time based on different selected independent variables. The trend change in health facility delivery was assessed using descriptive analysis stratified by various selected independent variables and assessed separately for each phase.

Multivariate decomposition analysis was used to identify the factors contributing to the change in the prevalence of health facility delivery over the study period. The decomposition analysis emphasis on how the change in health facility delivery responds to the differences in adolescent women’s characteristics and how these variables shape the changes across the survey conducted at different times. It is a regression analysis of the differences in the percentage of health facility delivery between EDHS 2005 and EDHS 2016. The aim of using the multivariate decomposition analysis is to identify the potential source of the differences in the percentage of health facility delivery in the last decades. The multivariate decomposition analysis for the non-linear response model uses the output of logistic regression analysis to divide the observed difference in the percentage of health facility delivery between the surveys into the components. The differences in the composition of the population (Endowment) and the difference in the effect of the characteristics (Coefficient) identify the factors contributing to the change in health facility delivery prevalence over time. The change in health facility delivery prevalence is additively decomposed into adolescent women's compositional change between the surveys (Endowment) and the difference in the effect of selected independent variables (Coefficient).

The recent EDHS 2016 and baseline EDHS 2005 surveys were denoted by A and B, respectively.

For logistic regression, the log-odds or logit of health facility delivery can be decomposed as:

$$\begin{aligned} & {\text{logit}}\left( {\text{A}} \right) - {\text{logit}}\left( {\text{B}} \right) = {\text{F}}\left( {{\text{X}}_{{\text{A}}} \upbeta _{{\text{A}}} } \right) - {\text{F}}\left( {{\text{X}}_{{\text{B}}} \upbeta _{{\text{B}}} } \right) \\ & \quad = \underbrace {{\left[ {{\text{F}}\left( {{\text{X}}_{{\text{A}}} \upbeta _{{\text{A}}} } \right) - {\text{F}}\left( {{\text{X}}_{{\text{B}}} \upbeta _{{\text{A}}} } \right)} \right]}}_{{\text{E}}} + \underbrace {{\left[ {{\text{F}}\left( {{\text{X}}_{{\text{B}}} \upbeta _{{\text{A}}} } \right) - {\text{F}}\left( {{\text{X}}_{{\text{B}}} \upbeta _{{\text{B}}} } \right)} \right]}}_{{\text{C}}} \\ \end{aligned}$$

where: E represents endowments explained by characteristics and C represents coefficients not explained.

We can rewrite the above equation as follow:

$$\mathrm{logit}\left(A\right)-\mathrm{logit}\left(B\right)=\left[{\beta }_{0A}-{\beta }_{0B}\right]+\sum {X}_{ijB}*\left[{\beta }_{ijA}-{\beta }_{ijB}\right]+\sum {\upbeta }_{ijB}*\left[{X}_{ijA}-{X}_{ijB}\right]$$

where; ${\beta }_{0B}$ is the intercept in the regression equation for EDHS 2005, ${\beta }_{0A}$ is the intercept in the regression equation for EDHS 2016, ${\beta }_{ijB}$ is the coefficient of the ${j}{th}$ category of the ${i}{th}$ determinant in EDHS 2005, ${\beta }_{ijA}$ is the coefficient of the ${j}{th}$ category of the ${i}{th}$ determinant in EDHS 2016, ${X}_{ijB}$ is the proportion of the ${j}{th}$ category of the ${i}{th}$ determinant in EDHS 2005, and ${X}_{ijA}$ is the proportion of the ${j}{th}$ category of the ${i}{th}$ determinant in EDHS 2016.

Currently developed multivariate logistic decomposition analysis for the non-linear response model used for decomposing the change in health facility delivery using **mvdcmp** STATA package [46].

## Results

### Background characteristics of the study population

In 2016 EDHS, the participants had an average age of 17.9 (SD ± 0.94) from age 15 to 19 years. Of the 378 adolescent women in the 2016, 146 (38.7%) women were delivered to their children in a health facility (public or private health facility), and 232 (61.3%) were not given birth in a health facility (Table 1). More Than three-fourth (72.3%) of adolescent women in three consecutive EDHSs aged 18 and 19 years. Based on residence areas, higher than ninety percent of adolescent women who had given a child residing in rural areas than their counterparts. Among adolescent girls, around eighty-three percent were married, and about (83.3%) of adolescent women were led by males. Regarding region, the highest percentage of adolescent women were from the large central region (Tigray, Amhara, Oromia, and SNNPs), and the smallest percent from the metropolitan region (Addis Ababa, Dire Dawa and Harari). Moreover, the proportion of all adolescent women who had given birth attended at least four ANC visits increased from 11% in 2005 to 30.6% in 2016; and approximately three-fourth (74.2%) of adolescent women have used ANC visit services (Table 1).

**Table 1:** Frequency and percentage of study participants for variables in Ethiopia for EDHS 2005, 2011, and 2016

Variables	Categories	EDHS 2005 N = 575 (%)	EDHS 2011 N = 492 (%)	EDHS 2016 N = 378 (%)
Place of delivery	Health facility	27 (4.6)	45 (9.2)	146 (38.7)
	Home	548 (95.4)	447 (90.8)	272 (61.3)
Maternal age	15–17	125 (21.7)	87 (17.7)	105 (27.7)
	18–19	450 (78.3)	405 (82.3)	273 (72.3)
Marital status	Single	20 (3.4)	36 (7.3)	23 (5.9)
	Married	514 (89.4)	409 (83.2)	308 (87.5)
	Separated/divorced	41 (7.2)	47 (9.5)	47 (12.5)
Religion	Orthodox	258 (44.8)	225 (45.8)	88 (23.1)
	Muslim	235 (40.9)	170 (34.6)	211 (55.9)
	Protestant	74 (12.9)	83 (16.7)	72 (91.1)
	Other	8 (1.9)	14 (2.9)	7 (1.8)
Sex of household head	Female	52 (9.1)	97 (19.8)	63 (16.6)
	Male	523 (90.9)	395 (80.2)	315 (83.4)
Maternal occupational status	Not working	458 (79.6)	279 (56.7)	227 (60.1)
	Working	117 (20.4)	213 (43.3)	151 (39.9)
Maternal education status	No education	443 (78.9)	252 (51.1)	124 (32.9)
	Primary	120 (20.9)	221 (45.0)	237 (62.6)
	Secondary and above	12 (2.1)	19 (3.9)	17 (4.5)
Wealth index	Poor	238 (41.4)	246 (50.1)	201 (53.3)
	Middle	140 (24.3)	114 (23.1)	89 (23.5)
	Rich	197 (34.3)	132 (26.8)	88 (23.1)
Living children	No child	43 (7.4)	27 (5.5)	18 (4.6)
	One	347 (60.4)	331 (67.3)	285 (75.5)
	Two and above	185 (32.2)	134 (27.2)	75 (19.9)
Use of ANC visits	No	323 (73.5)	242 (60.2)	88 (24.8)
	Yes	120 (26.5)	160 (39.8)	251 (74.2)
Frequency of ANC visits	No ANC visit	323 (73.5)	242 (60.2)	87 (25.8)
	1–3	68 (15.5)	101 (25.1)	148 (43.5)
	At least four visits	49 (11.0)	59 (14.7)	104 (30.6)
Place of residence	Rural	529 (92.0)	449 (91.3)	359 (95.1)
	Urban	46 (8.0)	43 (8.7)	19 (4.9)
Region	Large central	532 (92.5)	555 (92.6)	339 (89.9)
	Small peripheral	32 (5.6)	31 (6.2)	30 (7.8)
	Metropolitan	11 (1.9)	6 (1.2)	9 (2.3)

### Trends in prevalence of health facility delivery

**Fig. 1:**
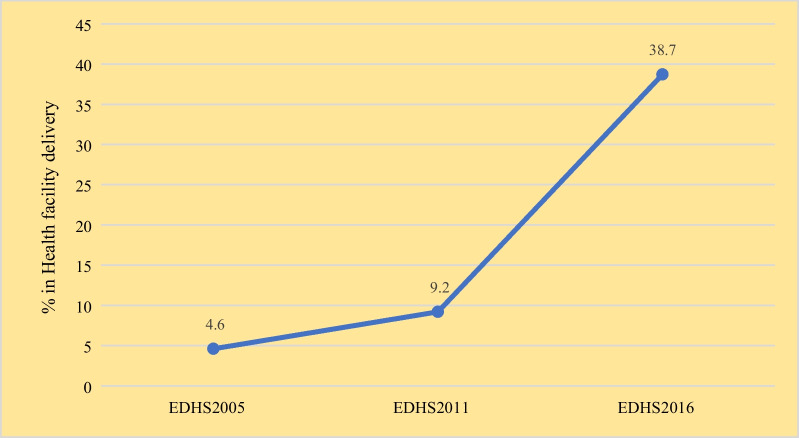
Trends of the health facility delivery among adolescent women in Ethiopia from 2005 to 2016

**Fig. 2:**
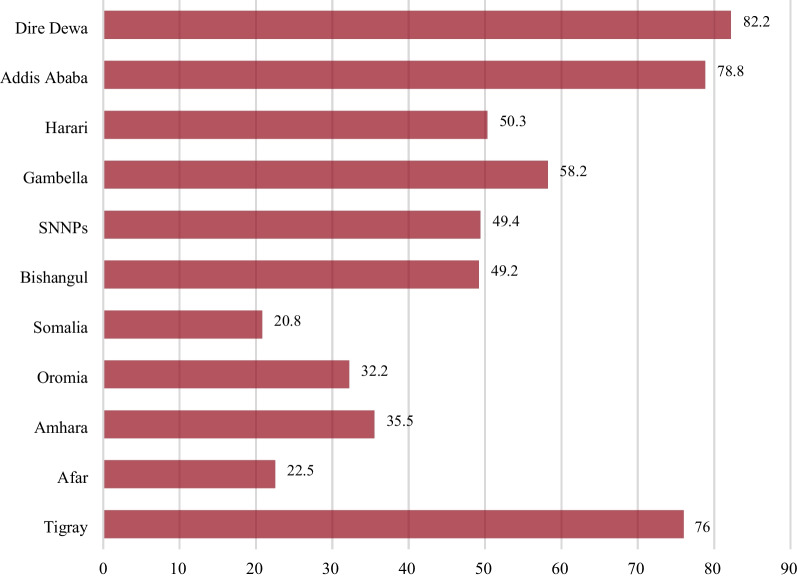
Prevalence of health facility delivery across regions in Ethiopia, 2016

In this study, the prevalence of giving birth in health facilities among adolescent women increased from 4.6% in 2005 to 38.7% in 2016. The 95% confidence interval for the percentage of adolescent women who had given a child in health facility delivery in Ethiopia increased from 4.6% (95% CI 3.2–6.7) in 2005 to 38.7% [95% CI 33.9–43.7] in 2016 (p-value < 0.001), with a 34.1% overall point change (Fig. 1). The prevalence of regional health facility delivery among adolescent women varied significantly overtime across regions ranging from 20.8% in the Somalia region to 82.2% in Dire Dawa (Fig. 2).

### Trends in the prevalence of health facility delivery by selected characteristics

Trends of health facility delivery showed variation based on different selected women’s characteristics. The percentage point change in health facility delivery increased in many categories of the variables in each phase (Table 2).

**Table 2:** Trends in health facility delivery among adolescent women by selected characteristics 2005, 2011, and 2016 Ethiopia Demographic and Health Surveys

Characteristics	EDHS2005	EDHS2011	EDHS2016	Percentage point difference in health facility delivery prevalence		
	N = 575	N = 492	N = 378	Phase1	Phase2	Phase3
				2011–2005	2016–2011	2016–2005
Health facility	4.6	9.2	38.7	4.6	29.5	34.1
Maternal age						
15–17	6.6	5.0	34.4	− 1.6	29.4	27.9
18–19	4.6	10.1	40.3	5.5	30.2	35.7
Sex of household head						
Male	4.3	7.8	37.6	3.5	29.8	33.3
Female	8.2	14.8	43.9	6.6	29.1	35.7
Place of residence						
Urban	36.7	74.6	82.1	37.9	7.5	45.4
Rural	1.8	3.0	36.5	1.2	33.5	34.7
Marital status						
Single	4.9	17.0	63.9	12.1	46.9	59.0
Married/living together	4.0	8.6	38.2	4.6	29.6	34.2
Widowed/divorced/separated	12.1	8.5	30.0	− 3.6	21.5	17.9
Religion						
Orthodox	7.2	11.4	48.0	4.2	36.6	40.8
Protestant	0.6	9.5	40.7	8.9	31.2	40.1
Muslim	2.9	6.9	35.3	4.0	28.4	32.4
Other	0.6	0.2	2.8	− 0.4	2.6	2.2
Maternal educational status						
No education	2.3	3.0	29.3	0.1	26.3	27.0
Primary	9.6	10.7	41.1	1.1	30.4	31.5
Secondary and above	38.9	73.5	74.8	34.6	1.3	35.9
Wealth index						
Poor	2.1	1.6	29.8	− 0.5	28.2	27.7
Middle	0.1	2.4	39.1	2.3	36.7	39.0
Rich	10.8	29.3	58.7	18.5	29.4	47.9
Maternal occupational status						
Not working	4.0	10.4	35.6	6.4	25.2	31.6
Working	7.2	7.6	43.4	0.4	35.8	36.2
Number of living children						
No child	0.1	9.3	20.4	9.2	11.1	20.3
First	6.3	11.7	43.9	5.4	32.2	37.6
Two and above	2.6	3.0	22.9	0.4	19.9	20.3
Frequency of ANC visits						
No ANC visits	3.9	2.6	18.2	− 1.3	15.6	14.3
< 4 ANC visits	4.9	20.4	39.7	15.5	19.3	34.8
At least 4 ANC visits	5.7	12.3	50.2	6.6	37.9	44.5
Distance to health facility						
Small problem	11.3	20.6	52.6	9.3	32	41.3
Big problem	2.1	5.8	31.2	3.7	25.4	29.1
Problem seeking permission to go healthcare						
Small problem	5.8	11.5	44.1	5.7	32.6	38.3
Big problem	2.5	3.9	33.4	1.4	29.5	30.9
Region						
Large central	3.2	9.0	38.7	5.8	29.7	35.5
Small peripheral	8.6	9.7	27.3	1.1	17.6	18.7
Metropolitan	61.3	21.1	74.9	− 40.2	53.8	13.6

The prevalence of health facility declined by a 3.9% point of change among rural residency during the third phase (2005–2016). Based on geographical regions, health facility delivery prevalence decreased in the last period (2005–2016) at a 3.8% point change in health facility delivery (Table 2). The highest point difference in the prevalence of health facility delivery was 53.8% in the metropolitan geographical regions (Harari, Addis Ababa, and Dire Dawa); and followed by 35.5% in the large central region (Tigray, Amhara, Oromia, and SNNPs) (Table 2).

According to maternal educational status, there was an increase in the percentage of health facility delivery among adolescent women with primary and secondary and above education in the last phase (2005–2016) by a 31.5% and 35.9% point change, respectively. Adolescent women who were married/or living together with their partners and Orthodox religious followers showed an increment in the prevalence of health facility delivery with 34.2% and 40.8% point change, respectively. Additionally, adolescent women who had attended at least four ANC visits showed an increment in health facility delivery prevalence with a 44.5% point change (Table 2).

### Decomposition analysis

#### Decomposition analysis of health facility delivery in Ethiopia, 2005–2016

In the overall trend analysis, there has been a significant increment in the prevalence of health facility delivery among adolescent women in Ethiopia. The decomposition results showed that an increment in health facility delivery prevalence over the study periods was explained by the difference in the selected independent variables and the effect of the characteristics between the two survey points (Table 3).

**Table 3:** Overall decomposition of the change in health facility delivery among adolescent women in Ethiopia, 2005–2016

Health facility delivery	Coef	[95% conf. interval]	Pct
Characteristics (E)	.26705	.13909 .39502	78.39
Coefficient (C)	.073617	.022014 .17765	21.61
Residual (R)	.34067	.26644 .4149	

#### Difference due to characteristics (Endowment)

Multivariate decomposition analysis results revealed that approximately 78.4% of the overall increment in health facility delivery was due to the difference in characteristics (the differences in the composition of adolescents’ selected variables) between the two survey points (Table 3). Multivariate decomposition analysis showed that place of residency, maternal educational status, wealth index, frequency of ANC visits, number of living children, and region were statistically significant variables for the change in the prevalence of health facility delivery. An increment in the composition of adolescent women who live in rural areas in the sampled population showed a significant inverse effect on the change in health facility delivery by 4.45%. An increment in the composition of adolescent women who have attended less than four ANC visits and at least four ANC visits contributed 6.16% and 14.88% to the change in health facility delivery prevalence, respectively (Table 4). The change in composition of adolescent women with secondary and above education over the study periods (from 2005 to 2016) showed a significant 5.73% positive contribution to the change in health facility delivery. An increment in the percentage of adolescents from wealthier showed a significant 15.48% positive contribution to the change in health facility delivery (Table 4). An increase in the composition of adolescent women having two or more living children showed a negative contribution to the change in health facility delivery by 4%. Moreover, adolescent women from metropolitan region in the sample showed a negative 5.1% contribution to the change in the prevalence of health facility delivery (Table 4).

**Table 4:** Detailed decomposition analysis of change in health facility delivery among adolescent women in Ethiopia, 2005–2016

Variables	Difference due to characteristics (E)		Difference due to coefficient (C)	
	Coef	Pct	Coef	Pct
Maternal age				
15–17^®^				
18–19	− .00040961 (− .010469 .0096498)	28.11	.10485* (.032497 .1772)	3.47
Place of residence				
Urban^®^				
Rural	.0064986* (.00066234 .012335)	− 4.45	.081709 (− .010276 .17369)	17.98
Maternal occupation				
Not working^®^				
Working	.0083153 (− .017219 .033849)	3.62	− .0024751 (− .02047 .015519)	− 3.8
Frequency of ANC visits				
No ANC visits^®^				
< 4 ANC visits	.014187* (.0034514 .024923)	6.16	.030483 (− .0073051 .068272)	8.95
At least 4 ANC visits	.057507 * (.0031008 .11191)	14.88	.0072807* (.0067122 .023274)	4.03
Maternal education				
No education^®^				
Primary education	.011341 (− .057104 .079787)	5.12	− .020115 (.043871 .0036406)	− 1.89
Secondary and above	.010534* (.0038693 .017198)	5.73	.0030437* (.0014446 .0075319)	2.9
Wealth index				
Poor^®^				
Middle	− .016479 (− .027181 .06014)	6.84	− .016298 (− .033896 .0012993)	− 2.63
Rich	.052725* (.0035259 .10192)	15.48	− .00051646 (− .0019098 .00087684)	− 1.24
Number of living children				
No child^®^				
First	− .0119 (− .058352 .034552)	− 0.78	.012211 (− .023826 .048247)	− 3.81
Two and above	.0094654* (.00081041 .01812)	− 4.0	− .016902 (− .047925 .081728)	− 2.96
Distance to health facility				
Small problem^®^				
Big problem	.0088187 (− .00032612 .017963)	3.58	.01515 (− .049002 .079302)	2.44
Seeking permission to go healthcare				
Small problem^®^				
Big problem	− .0036188 (− .023787 .016549)	− 4.13	.0090336 (− .022664 .040731)	2.65
Region				
Large central^®^				
Small peripheral	− .0064091 (− .01181 .010084)	− 2.87	− .0023921 (− .0057614 .00097728)	− 1.71
Metropolitan	.0036165* (.0012989 .0059341)	5.1	.000054574 (− .00070513 .00081427)	− 2.77

^®^: Reference category; *: Significant; Pct: Percentage; Coef: Coefficient

*Region is categorized as (Tigray, Amhara, Oromia, and SNNPs), metropolitan geographical regions (Harari, Addis Ababa, and Dire Dawa), and semi-peripheral region (Afar, Somali, Binshangul-Gumz, and Gambela)

#### Difference due to effects of the coefficient (Effects of characteristics)

The decompositional analysis showed that around 21.6% of the overall increment in the percentage of health facility delivery was due to the differences in the effect of the independent variables (coefficients) (Table 3). Factors including maternal age, maternal educational status and frequency of ANC visits showed a significant effect for the change in the percentage of health facility delivery. Compared with adolescent women aged between 15 and 17, the behavioral change of adolescent women who aged between 18 and 19 contributed 3.47% for the increment of health facility delivery prevalence over time. The change in behavior of adolescent women with secondary and above education contributed 2.9% for the observed change in health facility delivery over time (Table 4). Similarly, behavioral change of adolescent women who attended at least four ANC visits contributed 4.03% for an increment of health facility delivery.

Notice that the negative signs in the percentage of health facility delivery indicate a negative or reversal effect on the change in health facility delivery.

## Discussion

Health facility delivery is crucial to improving the health of maternal and newborns by providing safe and quality obstetric care [5]. In this study, the prevalence of health facility delivery among adolescent women in Ethiopia increased from 4.6% in 2005 to 38.7% in 2016. This finding is comparable with a study done in Ethiopia [35, 47]. This finding might be due to the launching of the Health Extension Program (HEP), improving access to health care to meet the primary attention of the SDGs agenda and the introduction of integrated community cause management program [48, 49]. It would be the increment of private medical facilities, which provide essential maternal health care services to practice delivery child in the health facility. However, institutional delivery rate among adolescent women was lower due to differences in access to health care services and disparity in the availability of health care services. According to this study, place of residency, maternal education, wealth index, frequency of ANC visits, number of living children, and region were statistically significant factors contributiong to give birth at health facility. On the other hand, maternal age maternal education, and frequency of ANC visits were a significantly factors contributing to women’s behavioral change in health facility.

The frequency of ANC visits is a significant factor contributing to health facility delivery among adolescent women. Maternal education level, maternal age, wealth index, place of residence, region, and the number of living children were also factors contributing to health facility delivery. The number of ANC visits had a statistically significant effect on health facility delivery among adolescent women. Adolescent women who had frequently attended ANC visits were more likely to give birth at a health facility than those who had no ANC visits. Those women who had attended the ANC visits repeatedly before giving birth may have various benefits for improving maternal health due to early identifying and managing adverse conditions upon pregnancy. It is consistent with the findings in other studies conducted in Ethiopia, Kenya, and Bangladesh among childbearing women [50–53]. This study noted that utilization of ANC visits is positively associated with childbirth at the health facility. An adequate number of ANC visits may encourage pregnant women to seek institutional delivery over time, which will increase their awareness of the possible complex and safe delivery practices [52, 54]. However, counseling services during the ANC visits were weak in various settings, but ANC visits were efficient in improving childbirth at health facilities [55]. Another possible reason for the increased use of health facility delivery among adolescent women might be that they may understand or be aware of the adverse consequences of pregnancy, which is an ongoing campaign to reduce the pregnancy rate and enhance the maternal health of women [8, 56]. However, this may also be that women are often like to give birth in any medical health facility as they are faced with a tremendous risk of delivery difficulties during childbirth.

Maternal education level is a significant factor that affects giving birth at the health facility. This finding is consistent with other studies done in Ethiopia and Nepal [35, 37, 38, 57]. Adolescent women with secondary or higher education have more knowledge about maternal health, and they are more aware of maternal health care services, including health facility delivery [13, 58]. In addition, studies conducted in Ethiopia and Nepal showed that adolescent women with secondary and higher education were more likely to make independent decisions about their health and well-being [59, 60]. Adolescent women with secondary or higher education could have good knowledge about health problems, and they use health care services effectively to achieve good health outcomes.

Different studies showed that place of residence had a statistically significant factors contributing to childbirth at health facilities among adolescent women, which was also identified in other studies [51]. An increment in the proportion of adolescent women who live in rural areas showed a negative contribution to the change in health facility delivery. Previous studies have shown that accessibility and high-quality health care services can affect birth at health facilities [55, 61, 62]. The increased rate of giving birth at the health facility in urban areas may be due to easy access to maternal and child healthcare services and better availability of medical care services in urban areas compared to rural areas [13, 58]. The behavioral change of adolescent women aged between 17 and 18 had a signifcant contribution to the change in health facility. This finding is supported with the study done in Nepal and Bangladesh [38, 63], they are more likely to seek skilled maternal health services and more aware of the consequences of pregnancy and childbirth. In addition, the possible reason could be the governments commitment to improve awareness of society through health education to usematernal healthcare services.

Similarly, this study also showed that wealth index status had a strong positive significant effect on birth at health facilities [51]. Various studies have shown that financial problems were the main challenge for adolescents in deciding where to give birth. Although the Ethiopian government subsidizes institutional delivery costs, wealthy adolescent women were more likely to give birth at a health facility than poor women; they could be able to cover financial payments related to institutional delivery like medicines and transportation [64]. As a result, the wealth index could have the ability to influence where adolescent women deliver their children. Besides, the number of living children is one of the statistically significant variable of health facility delivery among adolescents. This finding is consistent with other studies done in Bangladesh, Nepal, and Ethiopia [38, 51, 65]. The numer of living children in the household had a negative contribution to the change in the prevalence of health facility delivery. Adolescent women with two or more living children showed an inverse effect on giving birth in a health facility compared to those who had no living children. Adolescent women who had given two or more living children could not offer special attention to maternal health care services in health facilities because they have no previous pregnancy or childbirth complications and more responsibilities to care for their families [38]. When women did not experience child deaths, women might continue to give birth at home for the next childbirth, even if those women are aware of the benefits of giving birth at the health facility. This finding is comparable with other study findings [53, 54, 66]. As the number of living children in the household increases, women may not go to any medical health institution to give birth since they may have less time and higher responsibilities to maintain their families.

The principal strength of this study was based on the information of the national representative data set in Ethiopia. However, the weakness of this study was EDHS did not include all relevant information concerning accessibility (for instance, distance to a health facility) and available quality of health care services, which could affect childbirth at health facilities among adolescent women. We hope that these findings will be helpful and relevant for program designers and policy-makers to scale up the extent of health facility delivery and implement interventions to improve maternal health among adolescent women in Ethiopia. This finding does not address any programmatic and socio-cultural factors that could affect health facility delivery.

## Conclusions

The prevalence of health facility delivery had shown a significant inrease over the last decade years. Compositional changes in adolescent women's selected characteristics like the place of residency, maternal education status, frequency of ANC visits, wealth index, number of living children, and region were significant factors contributing to an increment in health facility delivery. Maternal age, maternal education, and frequency of ANC visits have contributed to the increment in health facility delivery over time due to the change in women’s behavioral changes.

It suggested that the increased efficiency of the health care system within society suffers from adolescent women's characteristics. Increasing women’s awareness of healthcare services utilization is highly recommended. Therefore, the government better focus on giving more attention to rural residents and poor women to give birth at health facilities.

## Data Availability

The EDHS dataset is publicly available, and you can access the data from the Measure DHS website (www.measuredhs.com).

## Abbreviations

ANC: Antenatal Care
CI: Confidence interval
EA: Enumeration Area
EDHS: Ethiopian Demographic and Health Survey
HEP: Health Extension Program
SDGs: Sustainable Developmental Goals
USAID: U.S.Agency for International Development
WHO: World Health Organization
